# Innovatively Supporting Teachers’ Implementation of School-Based Sex Education: Developing A Web-Based Coaching Intervention From Problem to Solution

**DOI:** 10.2196/jmir.5058

**Published:** 2016-07-12

**Authors:** Lisette Schutte, Marieke van den Borne, Gerjo Kok, Suzanne Meijer, Fraukje EF Mevissen

**Affiliations:** ^1^ Maastricht University Department of Work and Social Psychology Faculty of Psychology and Neuroscience Maastricht Netherlands; ^2^ STI AIDS Netherlands Department of Youth Amsterdam Netherlands

**Keywords:** implementation, web-based coaching, intervention mapping, sexual education, unobtrusive, fidelity, teachers

## Abstract

**Background:**

Full program implementation is crucial for effectiveness but is often overlooked or insufficiently considered during development of behavioral change interventions. For school-based health promotion programs, teachers are key players in program implementation, but teacher support in this phase is mostly limited to technical support and information. To ensure optimal implementation of the Dutch school-based sexual health program Long Live Love, a Web-based coaching website was developed to support teachers in completeness and fidelity of program implementation.

**Objective:**

The aim of this paper is to provide insight into the process of systematic development of a Web-based coaching intervention to support teachers in their implementation of a school-based sexual health program.

**Methods:**

The intervention mapping (IM) protocol was applied for the development of a theory- and evidence-based intervention. The IM process begins with (1) a needs assessment, followed by (2) the formulation of change objectives, (3) the selection of theory-based intervention methods and practical applications that take the parameters for effectiveness into consideration, (4) integration of practical applications into an organized program, (5) planning for adoption, implementation, and sustainability of the program, and finally, (6) generating an evaluation plan to measure program effectiveness.

**Results:**

Teacher’s implementation behavior was characterized by inconsistently selecting parts of the program and not delivering (all) lessons as intended by program developers. Teachers, however, did not perceive this behavior as problematic, revealing the discrepancy between teacher’s actual and perceived need for support in delivering Long Live Love lessons with completeness and fidelity. Teachers did, however, acknowledge different difficulties they encountered which could potentially negatively influence the quality of implementation. With the IM protocol, this Web-based coaching intervention was developed based on a concept of unobtrusive coaching, by and for teachers, to bring about change in teachers’ implementation behavior.

**Conclusions:**

This paper provides an example of a Web-based intervention to bring about behavioral change in a target group of intermediaries who lack intrinsic motivation for coaching and who’s perceptions differ from their actual problematic behavior. The IM protocol is a useful tool for guiding the scientific development of interventions and making them compatible with the needs and preferences of the target group.

## Introduction

Schools provide the ideal setting to reach youngsters with health promotion programs. Although decisions to use programs in schools are typically made at the administrative level, teachers are the primary agents of school-based prevention efforts. Their support, motivation, and commitment are crucial to implementation success [1]. In the Netherlands, teachers are the ones who decide to use a school-based program in their classroom (adoption), deliver the program to students (implementation), and continue to do so in the long run (continuation) [2]. Many evidence-based programs consider the implementers of the programs, such as teachers, as “core” to the success of the program [3].

Implementation of school-based health promotion programs is, however, not optimal [4,5,6]. These programs are not implemented with sufficient strength and fidelity to produce measurable outcomes [7]. A monitoring of school-based interventions in the Netherlands showed that only “5%-10%” of teachers who have bought a program, implement it fully in accordance with the ideas of the program designer, resulting in reduced program effectiveness [8]. The behavior of program implementers is often an aspect that is overlooked or insufficiently considered in program development. There is a need for greater attention for quality of implementation [9].

Implementation is a process consisting of several phases, namely adoption, implementation, and continuation [10]. Teachers need support in every phase of the implementation process to enable them to effectively carry out the program in their lessons [11,12]. Most interventions were aimed at supporting teachers in the awareness and adoption of the program but little is known or created to support teachers in the implementation phase [4,13,9,14]. Support in this phase is crucial, however, for optimal program effectiveness [13].

Especially when it comes to providing school-based sex education, delivering such lessons is not a simple or obvious task; teachers, who are key to the success of such programs, not only require knowledge and a positive attitude but also certain skills and competencies to deliver a range of sensitive topics in these lessons and to deal with the difficulties encountered during implementation of the program. To prepare teachers for program use, specialized and effective training is necessary [15]. Although training often equips teachers with skills for correct implementation, it is not enough [16]. It remains important to provide teachers with more personal assistance and ongoing support and consultation during program delivery to ensure the quality of implementation [6,17,18,14]. This support needs to be of sufficient duration to achieve depth in teachers’ skills and behavioral change throughout program delivery [19]. Paulussen et al [2] highlighted the importance of providing support before and during the implementation of a curriculum by way of training and technical and didactic assistance to ensure enduring success.

### The “Long Live Love” Program

In the Netherlands, Long Live Love (LLL) is the most widely used, effective school-based sexual education program, partly due to a successful dissemination strategy [20]. An earlier study on the implementation of LLL revealed that trainings from an external party, the Municipal Health Services (MHS), resulted in improved implementation of LLL by teachers [21]. Due to economic cutbacks, the supportive role of the Dutch MHS has recently been limited to predominantly stimulating dissemination and adoption of LLL and preparing teachers for initial implementation. They lack the capacity to provide intensive and long-term support [6]. In addition, MHS professionals lack the didactic expertise and skills to be appropriate role models for teachers in teaching skills for adequate implementation [22]. Teachers therefore need another form of support during implementation to compensate for the limitations of the MHS and to complement the existing dissemination strategy of LLL.

To contribute to the limited documentation of implementation interventions, this paper presents the systematic development of a Web-based coaching intervention, Lesgevenindeliefde.nl (teaching love). The website is part of a broader dissemination strategy and supports teachers in implementation of the school-based sexual education program, LLL. The Web-based coaching intervention aims at an optimal implementation, with completeness and fidelity, of LLL by teachers. As of date, no other Web-based coaching website to support teachers in delivering school-based sexual education is known in the Netherlands [23,24]. This paper will provide insight into teacher implementation of a school-based sex education program, LLL, and describe the complete cycle of development of this coaching website, from problem to solution. The website is developed applying intervention mapping (IM), a protocol to systematically develop interventions using theory and empirical evidence [25]. IM has proven to be an effective protocol in the development of various Web-based health promotion interventions [26,27,28].

## Methods

### Developing Effective Behavior Change Interventions

**Figure 1:**
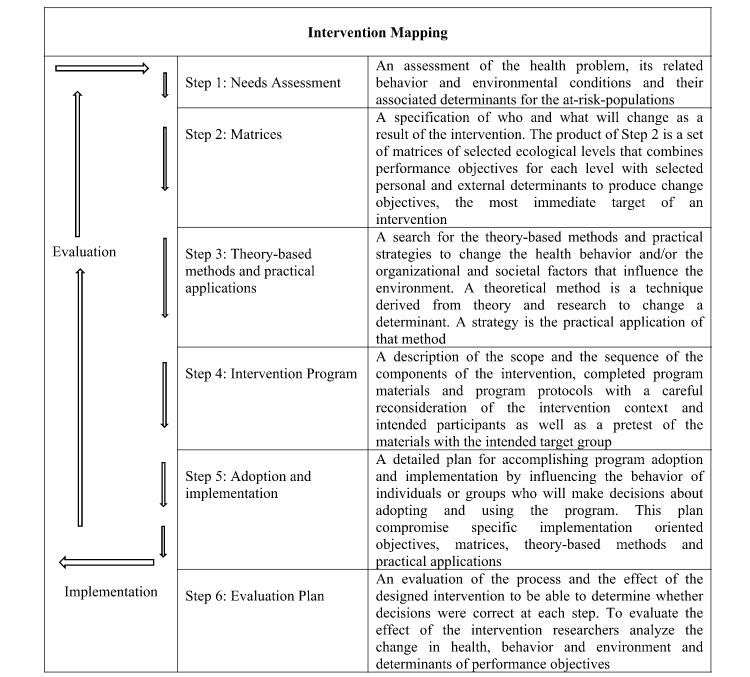
Intervention mapping process. Adapted from Bartholemew et al [25].

Intervention Mapping (IM) is a protocol for the development of theory- and evidence-based interventions. It maps the path from identification of a problem to the development of a solution. Although IM is presented as a series of 6 steps (see Figure 1), it is an iterative and cumulative process in which, respectively, the developer moves back and forth between the steps and in which each step is based on the outcomes of the previous ones [25]. The 6 steps are (1) conduct a needs assessment, (2) create matrices of change objectives, (3) select theory-based methods and practical applications, (4) organize methods and applications into an intervention program, (5) plan for adoption, implementation, and sustainability of the program, and (6) generate an evaluation plan [25].

### Intervention Mapping Steps

The first step, the needs assessment, begins with establishing a participatory planning group, represented by potential program participants and implementers. This step consists of a full analysis and description of the problem through an epidemiologic, behavioral, and social analysis of the at-risk-group. By means of qualitative and/or quantitative research, behaviors and environmental factors related to the health problem are identified.

In step 2, a transition is made from the problem to the solution, namely specifying what should change to prevent or to minimize a problem. Step 2 begins with the formulation of the behavioral and environmental outcomes to be achieved by the intervention followed by a breakdown of these outcomes into specific sub-behaviors called performance objectives, stating what the target group needs to do to achieve these desired outcomes. Next, determinants are selected that are linked to these objectives. Finally, these determinants and performance objectives are connected in a matrix to create change objectives, which state the specific goals to be achieved as a result of the intervention.

In step 3, theoretical methods are selected that change the specified determinants and achieve the change objectives. A method is a general technique for influencing change in determinants. These methods are translated into practical applications while taking the parameters for use into consideration. These parameters provide conditions under which effectiveness of the application is ensured. The applications should fit within the context of the intervention and the target group.

In step 4, creative and effective program components and materials are developed based on the previous steps. The challenge is to cover all selected theoretical methods, practical applications, and change objectives. The end product of this step is a coherent program that remains true to the planning that has been accomplished in step 1, 2, and 3.

Effective programs, however, will have limited impact if they are never, incorrectly or incompletely used. An appropriate adoption, implementation, and maintenance plan is essential to achieve the desired outcomes. The main goal of step 5 is to ensure that the intervention will be used and maintained over time for as long as is needed. To realize this goal, the developer must complete the same tasks as in step 1, 2, 3, and 4, resulting in an effective intervention plan for optimal adoption, implementation, and continuation of the intervention.

In the final step of the iterative and cumulative IM process, the effect and implementation of the intervention are evaluated. An evaluation gives insight into the extent to which the earlier formulated goals are achieved after application of the intervention. The evaluation is divided in outcomes of quality of life, health, and behavior. A process evaluation is necessary to understand these outcomes and gives insight in the “black box” underlying the effect. The “black box” provides insight into what happens between application of the intervention and the outcomes. This paper presents outcomes of steps 1-5. The effect and process evaluation will be presented in a separate paper.

## Results

### IM Step 1: Needs Assessment

At the start of the project, a participatory planning group was set up, consisting of a panel of health promotion professionals (N=10), teachers (N=4), and MHS professionals (N=2). The goal for the selected group was to think along in the intervention development process and be consulted throughout the project. The needs assessment was conducted by means of (1) analyzing existing programs and reviewing the literature and (2) qualitative research.

#### Analyzing Existing Programs and Literature Study

The search for existing programs in the Netherlands did not reveal the existence of systematically developed and evidence-based Web-based interventions for coaching teachers in providing sexual reproductive health (SRH) lessons. The search did result in a few materials to support teachers in teaching SRH. This support was, however, minimal and not aimed at coaching to bring about behavioral change. In the field of sexual health promotion, for example, there is a website for teachers, but this is limited to providing materials and practical information on how to provide such lessons without further coaching [29]. This is insufficient for behavioral change, which is necessary for completeness and fidelity of program delivery [25,30,12].

The literature study revealed that limited examples are available on the development, execution, and evaluation of implementation enhancing interventions in general. In fields other than SRH, studies were also mainly focused on the provision of technical support [31,32,33,34,35,36,37,38]. These studies, however, were not aimed at coaching to bring about behavioral change. Although related to themes other than SRH, these studies reconfirm the limited existence of evidence-based coaching interventions and emphasize the importance of systematically developing an intervention to accomplish sustainable behavioral change. Supporting teachers during implementation will enable them to deliver the lessons as complete as possible (completeness) and according to previously formulated program goals (fidelity) for optimal effectiveness [11,12].

#### Qualitative Research

Qualitative data were collected by conducting semistructured interviews with teachers to provide more insight into their implementation behavior and to get insight in their (perceived) need and preferences for coaching. A sample of 15 teachers from 12 different schools was selected from the database of schools who had bought the previous LLL program. The selection was made based on regional representation and gender. Teachers were asked to participate in the research by email. Furthermore, during the interview process, the snowball effect resulted in the involvement of 3 additional teacher respondents. The main reason for nonresponse was a lack of time. In the end, N=11 teachers signed up to participate for the interviews.

A topic list guided the interviews with 11 teachers (5 male, 6 female) from 9 different schools and regions, with diverse levels of experience in teaching SRH. The average duration of the interviews was approximately 40 minutes. See Textbox 1 for the topic list. This topic list was derived from a conceptual model based on the Theory of Planned Behavior [39], the Social Cognitive Theory [40], and the Diffusion of Innovations Theory [10]. These theories are often used to explain implementation behavior of teachers [21,2].

**Topic list needs assessment: Lesgevenindeliefde.nl**

- What do you do with the theme of SRH?
- How do you teach your SRH lessons?
- Do you have any idea how other colleagues are dealing with the theme of SRH?
- Do you have any idea how less experienced colleagues are teaching SRH?

- Which difficulties do you experience in teaching SRH?
- How do you deal with those difficulties?
- How can you address those difficulties?

- What do you need to be able to teach SRH optimally?
- What do you need to be able to effectively teach Long Live Love (LLL)?

- How do you prepare your SRH–lessons or for teaching the Long Live Love program?
- Do you use any kind of support or a program during the application of the LLL program or the SRH lessons?
- What do you do in the evaluation of the SRH-lessons or the Long Live Love program?

- Which support would you like to receive in teaching SRH?
- Which support would other, and maybe less experienced, colleagues like to receive in teaching SRH?
- How should this support or coaching look like?
- How can this be implemented in an internet based coaching program?

The interviews revealed that teacher implementation of SRH programs, including LLL, is not optimal; various components of the program are selected and delivered, rather than completing the entire program and implementing it as intended by the program developers. Teachers describe their implementation behavior as making a selection of program components, adjusting the program with their own additions, not delivering the program in its entirety, limited use of the teacher manual, and a lack of planning, preparation, and evaluation. This suboptimal implementation behavior may lead to reduced program effectiveness [4,5].

T: “When we teach Long Live Love, we sometimes make our own additions and modifications. The program lacks practical assignments. It’s mainly about reading and answering questions.”

I: “What is required to provide SRH programs optimally?”

T: “More practical materials. Actually, teaching SRH is mainly reading and answering questions. We do improvise with other materials because the SRH program alone does not contain sufficient practical assignments.”

Teachers do not acknowledge their behavior as problematic; they do not see the importance of delivering the lessons with completeness and fidelity for achieving program effectiveness and therefore expressed minimal need for coaching. Although the perceived need for support in implementation was low, teachers did recognize several difficulties that may be encountered, especially, according to them, by less experienced colleagues, during the provision of SRH lessons.

T: “Some colleagues, not myself of course, experience difficulties in talking about sexuality. How do you begin? How are you going to talk about it or cope with it? Coaching could be given for those kinds of problems.”

An inability to adequately deal with these difficulties can negatively interfere with optimal implementation of SRH programs. According to the respondents, teachers providing SRH lessons may encounter the following difficulties: (1) barriers to creating a safe and trusted atmosphere in the classroom; (2) feelings of shame or a closed attitude toward sexuality; (3) dealing with personal questions asked to them or to other students; (4) coping with individual student problems related to SRH; (5) dealing with homonegative reactions and behavior of students in the classroom; (6) anticipating on negative events on social media and Internet among students in relation to SRH; and (7) providing SRH lessons in a culturally, gender-wise, and sexually diverse classroom.

I: “What skills, knowledge, or other factors do you need to educate the students about SRH?”

T: “You have to perceive the world as students do. You shouldn’t be surprised by comments in the classroom. You shouldn’t assume that they are not sexually active. But you also need skills to create a safe and secure atmosphere for the students. They also have to be able to talk freely about their experiences. That you can use these experiences to give information and to integrate this in the lessons. It is also really important that there is respect for each other and for each other’s boundaries. That you are able to establish your boundaries. That you can be different. That’s also an important focus of our lessons.”

When teachers were asked what was necessary for effective implementation of SRH programs, teachers mentioned a desire for materials they could use in the classroom with their students. They were not focused on their own quality of implementation but instead they were on the lookout for practical tools to use during lessons.

T: “Teachers don’t often place their problems on a forum after a lesson. They will use it to find ideas for their lessons. Then they search tediously. But they won’t share the experiences they had during a lesson on a forum.”

I: “How can we optimally coach a teacher so that he is capable of teaching Long Live Love or SRH?”

T: “For teachers, it is necessary to be well informed about the topic. To have adequate, sufficient, and reliable information available. That they have the feeling; “I can answer questions.” That’s important in my opinion.”

I: “What more do teachers need?”

T: “Materials and good information. Ehm… Something to visualize. The classical cucumber with a condom.”

When the teachers were directly asked about their need for coaching, the respondents answered to be satisfied with their own teaching method and expressed minimal need to be coached. They felt they could prepare sufficiently by reviewing the program materials and the teacher manual individually, or were incidentally assisted by other teachers in preparations for program implementation.

T: “Well, in my case, I don’t know if I would use it (an e-coaching website) extensively. Because I have been teaching this (SRH) for a long time, I know a lot and everything can be talked about. If I don’t know something, I go to a colleague. So, I don’t know if I would make use of it. I would take a look. Purely out of curiosity. Maybe I am too arrogant but I really can’t think of anything I would need help for.”

Critical reflection of one’s own behavior and working on professional development are necessary for creating awareness and establishing sustainable behavioral change [41]. Teachers do not seem to see the connection between completeness, fidelity, and program effectiveness, do not see their suboptimal implementation behavior as problematic, or are not aware of potentially challenging situations and therefore do not see a need for behavioral change and coaching. Teachers need to be aware of the importance of completeness and fidelity in relation to program effectiveness, have insight in their (suboptimal) implementation behavior, and be aware of potentially challenging situations, to ultimately improve completeness and fidelity of program delivery. Critical self-reflection leads to awareness of own behavior and is the first step of coaching teachers toward behavioral change and professional development. Without a genuine recognition of need and desire, it is almost impossible to change behavior [41].

I: “Do you think teachers would make use of such a coaching website?”

T: “If you point out the things that can go wrong, they have to prepare to deal with them. If you can trigger that, you’ve already come a long way. Teachers will start to reflect; “how does that affect me?””

If a coaching intervention was to be developed, it is important, according to the teachers, to develop an intervention that is easy to use and accessible and does not cost a lot of time and effort because teachers claimed that they only have limited time and resources to prepare or to evaluate the lessons.

I: “How should such an e-coaching website look like?”

T: “There shouldn’t be any complicated access codes. A lot of people drop out if they see that. It should be easily accessible. It shouldn’t cost me an hour and a half to browse. I don’t have time for that. Ideally you can select various components on a website while browsing; difficult situations that you may encounter. If a recognizable situation is described by a fellow teacher, I might think, this can happen to me as well.”

The possibility of developing virtual coaching, in which teachers are guided throughout the implementation process by a virtual buddy, was discouraged by most of the respondents. Instead of a virtual buddy, teachers expressed the preference to communicate with colleagues within different schools to exchange ideas and teaching methods or to solve problems they encounter during the provision of SRH lessons.

I: “Do you evaluate or discuss your lessons?”

T: “No, it is a very lonely profession… It is progressive thinking to learn from other teachers.”

In conclusion, the needs assessment revealed an interesting finding: there is a discrepancy between teachers perceived and actual need for support in providing SRH lessons effectively. Teachers do not perceive their implementation behavior as problematic, but their actual implementation behavior does not fulfill the required completeness and fidelity for program effectiveness. To ensure fidelity and completeness of program implementation, it remains important to first create awareness, by means of self-reflection, of (1) the importance of completeness and fidelity in program implementation, (2) teachers’ current implementation behavior, and (3) the difficult situations they could potentially encounter. To achieve behavioral change, and contribute to professional development, teachers should subsequently be supported in dealing with the common difficulties mentioned and be provided with the knowledge and skills they need to implement SRH programs effectively. A careful choice for unobtrusive coaching techniques was made to ultimately bridge the gap between the perceived need and actual need of teachers for coaching. The technique of unobtrusive coaching is required to create awareness and accomplish behavioral change, despite the teacher’s resistance to coaching and ultimately optimize the role of the teacher in providing high-quality SRH lessons.

### IM Step 2: Matrices of Change Objectives

Based on the needs assessment and literature review, a program goal was formulated and subdivided into 4 desired behavioral outcomes for teachers. The program goal was that teachers in all secondary schools in the Netherlands implement LLL completely and according to its formulated goals (fidelity). The behaviors associated with this program goal were that teachers (1) reflect critically on and become aware of their implementation behavior regarding SRH, (2) implement LLL completely, (3) implement LLL according to guidelines in the teacher manual, and (4) deal adequately with difficulties that may be encountered during provision of SRH. These behaviors formed the outcomes of the intervention and were subsequently broken down into performance objectives. Performance objectives specified what teachers needed to do to perform those desired behaviors. The formulated behavioral outcomes and related performance objectives are presented in Table 1.

Performance objectives were then linked with their associated personal and external determinants. Determinants were specified based on the results of the needs assessment, a literature review and a review of theories. Social influence was not selected as a determinant because the interviews revealed that teachers individually determined their own method of teaching. However, skills, self-efficacy, attitude, and knowledge were found to be important determinants for teachers’ implementation behavior [6]. These determinants were evaluated on importance (how strongly is the determinant related to teacher’s performance objectives) and changeability (how easily can the determinant be influenced by a theory-based method), which formed the basis for the final selection of determinants that the intervention would target. A matrix was then created by combining the performance objectives and associated determinants to create change objectives; specific and measurable goals specifying what will change among teachers as a result of the intervention. For example, “teachers express confidence (determinant: self-efficacy) in creating a safe and secure atmosphere in the classroom when delivering LLL (performance objective)”. See Table 2 for a selection of change objectives.

### IM Step 3: Theory-Based Methods and Practical Applications

In this step, we selected theory-based methods to change the specified determinants and ultimately achieve the change objectives. These methods were derived from theories, predominantly the Social Cognitive Theory, Elaboration Likelihood Model, and the Trans Theoretical Model and from evidence in the empirical literature stating that the methods might have the desired effect to change the determinant [25]. The parameters, the conditions under which the methods were expected to be effective, were considered when translating them into practical applications, which fit within the context of the intervention and target group. Table 3 shows examples of selected theoretical methods, practical applications, and their relation to the selected determinants. For example, behavioral journalism is a potentially effective method for increasing self-efficacy but will only work under the condition that authentic interviews are used with actual community members, which represent the desired message [42]. This method was translated into the application of role model stories where teachers share their experiences and suggestions in dealing with difficult situations.

**Table 1:** Behavioral outcomes and performance objectives of teacher implementation.

Behavioral outcomes of teachers	Performance objectives
B.O.1. Teachers reflect and improve on their implementation behavior regarding SRH	P.O.1. Teachers reflect critically on their implementation behavior regarding SRH
	P.O.2. Teachers self-monitor and improve the weaknesses in their implementation behavior regarding SRH
B.O.2. Teachers deliver LLL to students completely (completeness=80% of program)	P.O.2.1. Teachers cover all 6 lessons of LLL
	P.O.2.2. Teachers use all program materials of LLL in each lesson (DVD, magazine, teacher manual, worksheets)
	P.O.2.3. Teacher covers the most important components of each lesson, as indicated in the teacher manual
B.O.3. Teachers deliver LLL to students according to the guidelines in the teacher manual (fidelity)	P.O.3.1. Teachers read the teacher manual as preparation for each lesson
	P.O.3.2. Teachers deliver each LLL lesson to students according to the teacher manual
B.O.4. Teachers deal adequately with the most common difficulties that arise during implementation of SRH	P.O.4.1. Teachers create a safe and trusted atmosphere in the classroom during all LLL lessons whereby students feel comfortable in the classroom and asking questions
	P.O.4.2. Teachers teach all themes in LLL without shame or taboos interfering with the quality of the lesson
	P.O.4.3. Teachers handle personal questions of students addressed to themselves depending on their personal need to answer these questions
	P.O.4.3.1 Teachers intervene whenever students ask them or fellow students questions that are too personal
	P.O.4.4. Teachers integrate the theme of homosexuality as self-evident during all lessons of LLL
	P.O.4.4.1 Teachers intervene on homonegative behavior of students
	P.O.4.5. Teacher handle cultural, gender, and sexual experience diversity in the classroom using an approach that address and involves all students
	P.O.4.6. Teachers identify individual problems of students with and refer them to the appropriate help
	P.O.4.7. Teachers address actual themes within social media and Internet in relation to SRH during the provision of LLL
	P.O.4.8. Teachers facilitate discussions in the classroom about relationships and sexuality according to the formulated goals and suggestions in the teacher manual

**Table 2:** Sample of change objectives

Homosexuality	Knowledge	Attitude	Self-efficacy	Skills
1. The teacher integrates the theme of homosexuality as self-evident during all lessons of Long Live Love	K 1.1 The teacher describes how homosexuality is integrated in the lessons of Long Live Love.	A 1.1 The teacher expresses the importance of a positive attitude of a teacher toward homosexuality during the application of the lessons of Long Live Love.	SE 1.1 The teacher expresses confidence in ability to replace “he” and “she” by “he” and “he” or “she” and “she.”	S 1.1 The teacher demonstrates how he/she continually integrates the theme of homosexuality in the lessons.
	K 1.2 The teacher lists the moments in the Long Live Love lessons where the theme of homosexuality can be discussed as a self-evident part of the lesson.	A 1.2 The teacher expresses the advantages of integrating homosexuality as self-evident during the application of Long Live Love.	SE 1.2 The teacher expresses confidence in the ability to continually integrate homosexuality and certainly not avoid the theme in the lessons of Long Live Love in case of negative reactions from students.	S 1.2 The teacher demonstrates skill to not avoid the theme of homosexuality despite possible adverse or negative reactions from students.
	K 1.3 The teacher describes how he/she plans to integrate homosexuality in the lessons of Long Live Love.	A 1.3 The teacher expresses the importance of mentioning “he” and “he” or “she” and “she” instead of “he” and “she” during the lessons of Long Live Love.	SE 1.3 The teacher expresses confidence in the ability to protect students with feelings of homosexuality against a feeling of discomfort or social pressure.	S 1.3 The teacher demonstrates skills to stimulate the discussions about homosexuality in which respect and acceptance are important key aspects in this in depth discussion.
	K 1.4 The teacher explains that when “he” and “she” is mentioned this can also be replaced by “he” and “he” or “she” and “she.”	A 1.4 The teacher expresses the importance of discussing and integrating the theme of homosexuality, especially when the students react negatively.	SE 1.4 The teacher expresses confidence in the ability to communicate the message that homosexuality is not limited to borders, cultures, or countries during the lessons.	S 1.4 The teacher demonstrates how he/she protects students with homosexual feelings from a feeling of discomfort.
	K 1.5 The teacher explains the reasons why he or she will strive toward a self-evident integration of homosexuality as theme in the lessons of Long Live Love.	A 1.5 The teacher describes the importance of effectively coping with feelings of pressure or discomfort of students with feelings of homosexuality during the lessons of Long Live Love.	SE 1.5 The teacher expresses confidence in the ability to be continually alert of possible individual confrontations between students about homosexuality.	
		A 1.6 The teacher expresses the importance of informing students that homosexuality is not limited to a culture, to borders, or to periods.		
				
2. Teachers intervene on homonegative behavior of students	K 2.1 The teacher lists the signs he/she should be aware of which could indicate homonegative ideas or behavior among students.	A 2.1 The teacher expresses a disapproving attitude toward homonegative behavior during the application of Long Live Love.	SE 2.1 The teacher expresses confidence in the ability to be continually alert of signs or behavior of students in the classroom, which can be homonegative.	S 2.1 The teacher demonstrates skills to constantly be alert of homonegative signs or behavior of students during the lessons.
	K 2.2 The teacher describes which methods can be used effectively in the classroom when students have homonegative ideas or show homonegative behaviors.	A 2.2 The teacher describes the importance of being constantly alert of homonegative signs or behavior of the students.	SE 2.2 The teacher expresses confidence in ability to take measures when students act homonegatively in the classroom.	S 2.2 The teacher demonstrates skills to adequately deal with homonegative signs or behavior of students in the classroom.
	K 2.3 The teacher describes how homonegative reactions of students can be used as a subject for discussion.	A 2.3 The teacher expresses the importance of taking timely measures when students act homonegatively in the classroom.		

**Table 3:** Methods and applications.^a^

Determinants	Methods	Parameters	Applications	How population, context, and parameters were taken into account
Knowledge	Elaboration	High motivation and cognitive ability, personally relevant messages, surprising, repeated, self-pacing, not distracting, easily understandable, include direct instructions	Informative texts, tips, and FAQ	*Population:* The informative text was derived from professional teacher channels as well as from teachers themselves and health promotion, didactic, and pedagogic professional information. *Context:* Texts and tips were included within each sub homepage for each difficulty. *Parameters:* The texts were revised by an editor, composed based on teachers’ experiences and relevant literature for the area of expertise.
	Feedback	Specific, follows behavior in time, individual	Email option and options to post reactions on role-model stories and films	*Population:* To be able to answer specific individual questions and provide individual feedback this function was integrated in the website. *Context:* In case of a direct coaching question from the visiting teacher, he/she was able to ask questions through a mail-function or post comments below a story or film. *Parameters:* The mail form was only accessible for the individual teacher. The question or answer was not published for others. Continuation in contact could be initiated by the teacher.
	Discussion	Listening to learner to ensure correct schemas are activated	Options to post comments on role-model stories and films	*Population:* Visiting teachers were stimulated by an open question to post comments. The open question structured the discussion. *Context:* The aim of the comments below the role-model stories and the film was to stimulate a discussion between visiting teachers about the topic discussed in the story or film. It also served as a platform for discussing tips on how to deal with that specific difficulty. *Parameters:* The placed reactions were visible for all visiting teachers. A Youtube-like structure was used for optimal usability.
Skills/self-efficacy	Behavioral journalism	Credible message, model gives reasons for adopting new behavior and states perceived reinforcing outcomes received	Rotating photo’s, role-model stories and films	*Population:* Interviews with teachers were used in several aspects of the website to realize a platform by and for teachers. *Context:* Photo’s and interviews were used to compose role-model stories, films, and photos. These stories and films were based on a structure in which first the problem is presented as well as the experience and the relevance of this problem followed by the search for the most effective solution with a description of failures and success factors. *Parameters:* The interviewed teachers were selected to present a diverse selection in teaching experience, in geographic location and personal characteristics and were coping models, instead of mastery models, to increase the identification.
	Modeling	Attention, remembrance, self-efficacy and skills, reinforcement of the model, identification with model, coping instead of mastery model, demonstrate relevant skills	Rotating photos, role-model stories, and films	*Population*:To create a platform for and by teachers, teachers were interviewed which formed the content for role-model stories and films. Photos of teachers were taken to increase reliability and credibility as well as to lure teachers to the website. *Context*:The interviews were used to fill in the main content of the website. *Parameters*:Interviewed teachers were selected on personal characteristics, on geographic location, and on experience to create a database of diverse teachers that the target group could identify with. The interviewed teachers were all coping models.
Attitude	Self-reevaluation	Feedback and confrontation; however, raising awareness must be followed by increase in problem solving ability and self-efficacy	Self-reflection tool	*Population*: Teachers expressed a minimal need for coaching but teachers’ implementation behavior was characterized by inconsistently selecting parts of the program and not delivering all lessons. To bring about behavioral change first a self-reflection intervention is necessary to create professional awareness as the first step of improving implementation behavior. *Context*:In this self-reflection tool teachers could score different aspects of their own professional behavior in dealing with difficulties on a Likert scale. *Parameters:* After completion of the self-reflection tool an overview of gaps in learning were revealed. The teachers were directly referred to the most personal relevant difficulties.
	Scenario-based information	Plausible scenario with cause and outcome, imagery	Role-model stories and films	*Population:* Teachers were interviewed to collect data and to form the content for the scenarios. Teachers were coping models who were also experiencing the same problems as the target group but also found a solution. *Context:* In the films and stories, interviewed teachers were especially asked to describe scenarios to make the learning process applicable in daily practice and for the individual situation of a teacher. *Parameters*: The scenarios were described according to a structure in which the (personal) relevance and description of the problem was made followed by a search for the most effective solution.
	Modeling (see above *)*			

^a^Bartholemew et al [25]

### IM Step 4: Program Development

In this step, the intervention program is designed and materials are developed. The intervention, called Lesgevenindeliefde.nl (teaching love), was designed as a coaching website for teachers.

Although teachers expressed a minimal need for coaching during the interviews in the needs assessment, the program developers nonetheless saw the need to develop Lesgevenindeliefde.nl for the following reasons: (1) quality of implementation by teachers is suboptimal, despite their conviction about their own teaching method. To change this conviction and improve teacher implementation behavior, self-reflection and professional development are necessary. Teachers do not usually reflect on their own implementation behavior. Self-reflection, however, could lead to a critical evaluation and subsequently to improvement of their implementation behavior, which ultimately contributes to an increased effectiveness of an intervention. Coaching can only start when teachers develop an awareness of the need and a desire to improve their performance or change the way they have been performing at work [41]. Confronting teachers with potential difficulties they could encounter might help them to reflect on their behavior. (2) Teachers could use support in adequately dealing with the difficult situations encountered when delivering SRH lessons, as mentioned in the needs assessment. This could lead to improved program implementation. (3) The development of a website is an efficient, low-threshold way of reaching a mass of teachers. It partly replaces and supports the implementation promoting tasks of the MHS, which now lacks the capacity and didactic expertise for training teachers. The choice of the Internet as a channel of the intervention was predetermined by the program financers.

To ensure that the intervention was appealing and trustworthy to teachers, the coaching website was based on the concept “by and for teachers,” with role model stories, photos, and videos as the main products of this concept. This concept was chosen because teachers stated that if they did seek support during the delivery of SRH lessons, they preferred to consult fellow teachers or considered other teachers as reliable sources of information and for seeking advice. A large study in the Netherlands found that teachers in secondary schools either consult colleagues in their school for information or use the Internet to find information, to prepare their lessons, send emails to students, or give homework assignments [23]. Studies in the United Kingdom pointed clearly to the value of teachers learning with and from each other when it comes to professional development [43,44].

Certain characteristics of the website such as its accessibility, usability, flexibility, and tailorability to needs of teachers potentially limited barriers to visiting the website. Teachers could flexibly access all the information on the website that was personally relevant rather than being forced through a fixed coaching program. This catered to their lack of time and diverse needs for support. Accessibility to the website was simplified by placing the link on the LLL e-learning website under the “teacher” button. Teachers were encouraged to come back to the website by constantly placing new updates there and by integrating teacher materials in the website.

The Web-based intervention was developed with the underlying idea of an unobtrusive coaching technique whereby teachers’ actual needs were addressed and their perceived need of coaching was changed. With this technique, we attempted to trigger teachers to become aware of their need for coaching, without awakening resistance, by exposing them to difficulties experienced by other teachers they identified with. To meet teachers’ need for student materials, teaching materials were provided via the website. This student material can function as the first trigger for teachers to visit the website but was strategically placed at the bottom of the navigation system to ensure that teachers were first exposed to the most common difficulties encountered during implementation. It is a necessity that teachers effectively cope with difficulties to prevent these from becoming a barrier to optimal delivery of the program. Subsequently, elimination of these barriers is followed by practical support in delivering the lessons to ultimately accomplish completeness and fidelity of the delivery of LLL.

Furthermore, teachers were lured into the deeper structure of the website by presenting clickable rotating quotes with photos of teachers they could identify with. This is in line with the concept “by and for” teachers derived for the method of peer coaching. Peer coaching suggests that the professional development of teachers can be improved through experimentation, observation, reflection, the exchange of professional ideas, and shared problem‐solving [45]. The information on the website was given by teachers instead of experts as they are coping models, not mastery models, which is important for the acceptance of the message [25].

**Figure 2:**
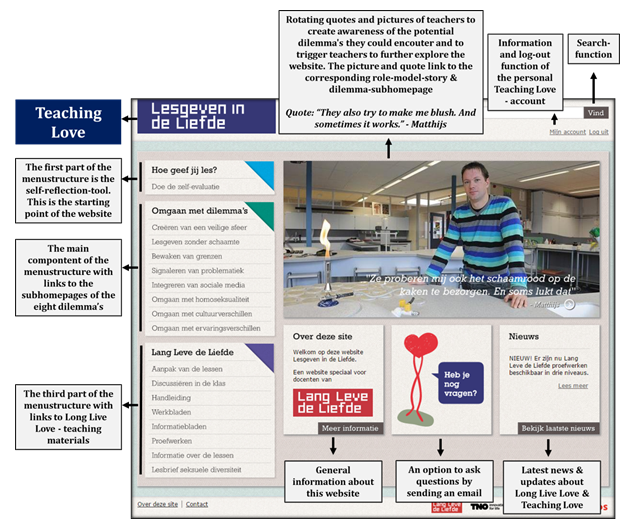
Homepage of Lesgevenindeliefde.nl.

The homepage of the coaching website includes a left-menu structure and rotating photos of teachers with SRH teaching–related quotes. These photos of teachers with short rotating quotes, placed in the center of the homepage, were meant to increase teachers’ awareness of the most common difficulties and to trigger their perceived need to be coached. These quotes also served as cues for teachers to browse further through the website and as an entrance to the related role model stories. Furthermore, on the homepage, general information about the website could be found as well as the possibility to ask for support via email, up-to date information about SRH, a search function, and frequently asked questions (see Figure 2). Additional subpages could be reached by clicking on any of the features presented on the homepage.

To access the website, teachers had to sign up with a self-created user name and password. The sign-up was included to protect the privacy of teachers and to provide a protected Internet environment where teachers could safely exchange their ideas. The website also needed to be secured to prevent students from accessing it. For the program developers, these account details provided demographic information about the users and the use of the website.

Access to the main content of the website was predominantly navigated through the left-menu structure. This structure contains three categories: (1) a self-reflection tool to trigger teachers to reflect critically on their implementation behavior, (2) the 8 main difficulties that teachers may face when implementing SRH lessons, each with their own underlying sub-homepage, and (3) student materials and practical instructions teachers need to implement LLL completely and with fidelity. See Figure 2 for an impression of the homepage.

The left-menu structure was chosen for usability purposes, mainly because it presented the website content and structure clearly. This made the website accessible and easy to use. Current practices strongly recommend placing the main navigation menu on the left-hand side of the page [46]. The choice of this navigation system addressed teacher’s limitations of time and skills in the Internet use and the general preference of website visitors to be able to have a clear navigation on the homepage [47].

**Figure 3:**
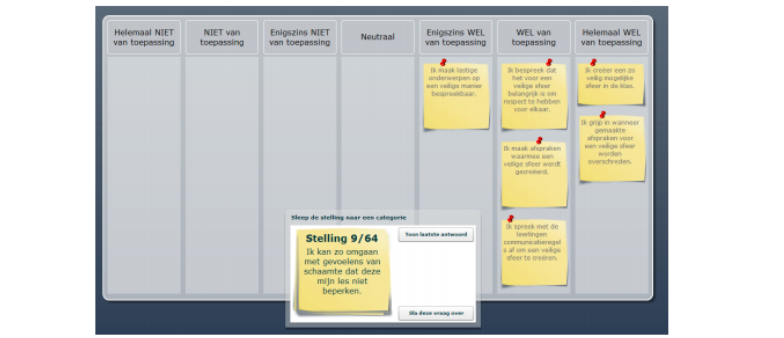
The self-reflection tool.

The first part of the left menu structure consisted of a self-reflection tool. This was developed to enable teachers to reflect critically on their own implementation behavior and to create awareness of their need for coaching concerning the most common difficulties in providing SRH. The self-reflection tool was developed in the form of an interactive questionnaire whereby each statement related to teaching SRH was rated on a Likert scale of 1 (totally disagree) to 7 (totally agree). For example on the difficulty of teaching SRH without shame, the following statement had to be rated from 1 to 7: “I can deal with feelings of shame in such a way that they do not limit my lesson” (see Figure 3). With the results of the reflection tool, teachers were referred to personally relevant pages on the website to enable them to improve these specific behaviors.

The central component of the left menu structure focused on the 8 main difficulties encountered during implementation by presenting them via 8 separate buttons, each with its own sub-homepage. The difficulties were (1) creating a safe and secure atmosphere for the provision of SRH lessons, (2) teaching SRH without shame influencing the quality of the lessons, (3) protecting boundaries in sharing personal information and questions between teachers and students and among students themselves, (4) identifying personal problems of students, (5) integrating social media in SRH lessons, and (6) integrating homosexuality as self-evident during the lessons of LLL and dealing with negative reactions to homosexuality and anticipating on (7) differences in culture, and (8) different levels of sexual experience in the classroom.

**Figure 4:**
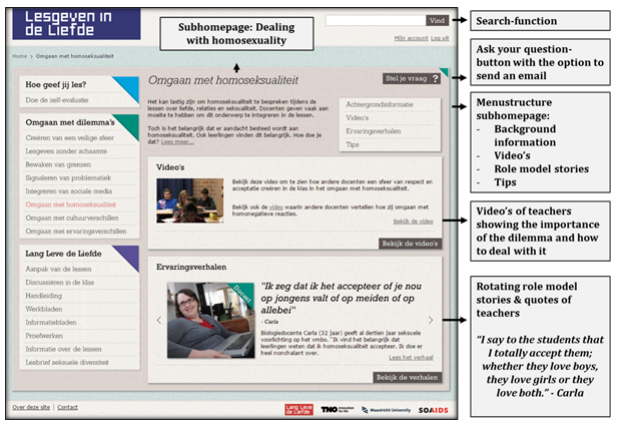
Subhomepage: Dealing with homosexuality.

By clicking on one of these menu items, the visitor entered a sub-homepage with background information on the specific difficulty, videos, role model stories with rotating quotes, and suggestions to adequately deal with that specific difficulty. The rotating quotes served the same function as the quotes on the homepage, namely to make them aware of the personal relevance of the difficulty, awaken their need to adequately deal with that difficulty and trigger them to access further content. Each role model story and video had the possibility of posting a comment, similar to existing commentary structures, such as YouTube (See Figure 4).

**Figure 5:**
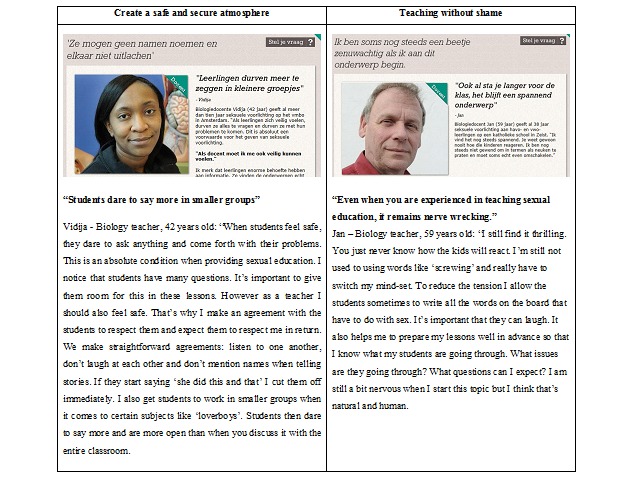
Examples of role model stories.

The content of the website was mainly obtained by interviewing teachers for role model stories and videos. It was important that the video’s and role-model stories were realistic; teachers had to be able to identify with them, the content had to be recognizable, and a clear solution on how to cope with the difficulty had to be provided. Each video and role model story was based on the structure of first introducing the teacher to the difficulty and then providing a solution. First knowledge, then awareness, attitude, and then skills were addressed in these videos and role model stories. The role model first introduced and acknowledged the difficulty (knowledge and awareness), then expressed the importance of dealing adequately with the difficulty (attitude) and finally described how (s)he effectively coped with the difficulty in such a way that it did not influence the implementation of LLL (see example of role model stories in Figure 5). The role model stories and videos were supplemented by general suggestions including didactic and pedagogic information and background information concerning the difficulty. This information was collected from websites and articles as well as from own research.

The last part of the left-menu structure was specifically aimed at effective practical delivery of the LLL program. Optimal delivery was achieved by informing teachers how to best provide LLL lessons and how to handle discussions in the classroom by means of role model stories and videos. Teachers could also download teaching materials such as the teacher manual, work, and information sheets for students and general information about the LLL curriculum. These materials were strategically included in the website to lure teachers to the website and meet the need they expressed in the needs assessment for such teaching tools.

### IM Step 5: Implementation

The coaching website (Lesgevenindeliefde.nl) had to be used by teachers to have an impact on program effectiveness and ultimately on student outcomes. In this step, an implementation plan was made to ensure that teachers were aware of the existence of the website and made use of it. Despite being designed to support teachers in their implementation of LLL, the website itself also needed to be effectively implemented.

The implementers of the coaching website are the program developers, STI AIDS Netherlands, who maintained, monitored, and updated the website and made it available and easily accessible on the Internet. Teachers were the end users of the website. The implementers developed dissemination tools, according to the IM protocol, to ensure that teachers were exposed to the website and to increase awareness and use of the website. An information brochure including information about LLL and the website was created. In addition, a trailer of the website was developed, explaining the aim of the website and showing the content and use of it (see Multimedia Appendix 1). This trailer and further information about the website were also integrated into the training provided to teachers by the MHS. In this training, teachers were informed by the MHS about LLL as well as the existence, advantages, and use of the coaching website, thereby stimulating teachers to use it when implementing LLL.

To further ensure teachers’ awareness of the website and ability to make use of it in an efficient manner, a link to the website was integrated into the teacher manual. In the manual, references were made to the website in each lesson where relevant or wherever a specific difficulty was expected to arise in that lesson. The aim and functionalities of the website were also described in the teacher manual.

### IM Step 6: Evaluation

In this step of IM, an evaluation plan and the corresponding evaluation measures were identified and developed. An effect and process evaluation for the pilot implementation of the coaching website, Lesgevenindeliefde.nl, was performed. This occurred simultaneously with the pilot implementation of the school-based LLL intervention for students [20]. Formulated outcomes of steps 2 and 5 of IM, namely the change objectives and the implementation goals, were used in creating the evaluation plan. The aim is to find out how and to what extent teachers made use of the website, how they appreciated it, what effects it had on their implementation of LLL (completeness and fidelity), and what factors affected teachers use of the website. This was investigated using a randomized controlled trial design. Qualitative and quantitative data were collected. Results of this study will be described elsewhere.

## Discussion

### Principal Findings

In this paper, the Intervention Mapping (IM) protocol was applied for the development of a theory- and evidence-based Web-based coaching intervention, Lesgevenindeliefde.nl (teaching love), aimed at supporting teachers in their implementation of the sexual education program, Long Live Love (LLL). The IM protocol is perceived as a useful tool for guiding the development of this intervention and making it one which is compatible with the needs and preferences of teachers. With the IM protocol, careful decisions were made using a cumulative and iterative process, resulting in this Web-based implementation support intervention by and for teachers. Although IM was useful in designing this intervention, it is also a time-intensive method, which was exacerbated by limited available resources in the project. Predetermined requirements of the program financers, such as budget and time, and available capacity and time of the MHS restricted the options for the type of intervention such as a digital versus a personal form of coaching. The method of IM used to develop the e-coaching intervention can be applied in other school settings or extrapolated to other areas of health promotion [25].

The first step in the IM process revealed an interesting and challenging discrepancy between teachers' actual implementation behavior and their perception of their behavior. Several studies revealed that there is an implementation problem among teachers but teachers themselves do not perceive this suboptimal implementation behavior as a problem [4,5,48,16,49]. In the case of LLL, teachers did not deliver lessons completely and according to the goals of program designers but teachers did not perceive this behavior as problematic or as a necessity to effectively provide the lessons. This behavior, however, could possibly result in decreased program effectiveness. Teachers did, however, acknowledge some difficulties encountered when providing sexual education, who according to more experienced teachers, were predominantly faced by less experienced colleagues. These difficulties could affect the fidelity and completeness if they are not adequately dealt with.

Teachers remain a difficult target group for bringing about behavioral change and made it challenging to develop a coaching intervention. Although the intervention was necessary, teachers expressed resistance and reluctance and experienced a limited intrinsic motivation to be coached, thereby rendering directive coaching as a noneffective method for behavioral change of this target group. Directive coaching has been found to be an effective method to expose teachers to the intervention content but eagerness is a necessity before entering and being involved in this directive coaching process [47]. Lack of intrinsic motivation namely forms a barrier to coaching [50]. Without a need and desire to be coached, it is almost impossible to change behavior. Coaching can only start when teachers develop an awareness of the need and desire to improve their performance or change the way they have been doing things at work [41]. This resulted in selecting the technique of unobtrusive coaching to create awareness without creating resistance; a nondirective way to bring about behavioral change. To achieve behavioral change, self-reflection is an essential first step to create intrinsic motivation to be coached and to ultimately develop professionalism in SRH. In addition, a concept of peer coaching was integrated, by and for teachers, to lower the resistance and to ultimately achieve behavioral change. Peer coaching suggests that the professional development of teachers can be improved through experimentation, observation, reflection, the exchange of professional ideas, and shared problem solving [45].

To lure teachers to the website, student materials were made available on the Web. The lack of intrinsic motivation to visit the website triggered program developers to invest in additional implementation activities because a website alone would be insufficient to involve teachers who are not intrinsically motivated. Integrating the website in the teacher manual, incorporating information about the website in teacher training, and developing a trailer to create awareness and enthusiasm among teachers for the website are examples of implementation activities.

The increased use of digital technologies in the education system, such as Lesgevenindeliefde.nl, brings exciting opportunities for innovative ways of teaching and learning. New, Web-based technologies do not only provide an anonymous communication space but also offer students and teachers easier, affordable, convenient, and faster access to information, teaching and learning resources, peers, experts, and a wider community. Web-based technology is also a low-threshold and efficient way of reaching many teachers and providing support in, for example, the implementation of school-based programs. Exploring the educational potential of these digital technologies and supporting schools in making optimum use of them remains important [51].

### Conclusions

With the development of the e-coaching website, a unique contribution was made in the field of bringing about behavioral change among intermediaries, especially due to the elements of self-reflection and unobtrusive peer coaching. The use of Web-based coaching to improve implementation behavior of teachers could be generalized to different cultural contexts because it addresses the common challenges faced in the area of sexual health education in schools worldwide [52]. Our process of intervention development may be applied to get from problem to solution regarding diverse implementation problems in development of interventions for challenging target groups. In addition, other health promoting professionals may benefit from our example of the ongoing process of balancing input of the target group with the wishes of the intervention developers to ultimately develop an effective intervention.

Lesgevenindeliefde.nl will be tried out in practice by means of a pilot implementation. During this pilot-implementation, the website will be evaluated on process and effect. Based on the experiences of teachers and outcomes of the studies, further enhancements of the website could be made. The introduction of an innovation, such as Lesgevenindeliefde.nl, could present certain challenges in the implementation phase. The process of accepting the innovation takes time, as described in Rogers’ diffusion curve [53]. The innovation will most likely be adopted by innovators first, followed by the early majority and eventually the laggards. Pijpers et al [54] claim that Web-based innovations first need to be accepted to be used broadly and effectively. The systematic approach and customized concept of Lesgevenideliefde.nl can serve as a distinct example of how to bring about behavioral change in a target group of intermediaries who lack intrinsic motivation for and have resistance to coaching. This approach can be applied to other SRH programs in the school setting and to target intermediaries in health promotion.

## Appendix

Multimedia Appendix 1 Trailer of ‘Lesgevenindeliefde.nl’.

## Abbreviations

IM: intervention mapping
LLL: Long Live Love
MHS: Municipal Health Services
SRH: sexual reproductive health
